# Association of dietary fats with ischemic heart disease (IHD): a case–control study

**DOI:** 10.1186/s41043-023-00489-w

**Published:** 2024-02-01

**Authors:** Mobina Zeinalabedini, Maryam Ladaninezhad, Khadijeh Abbasi Mobarakeh, Anahita Hoshiar-Rad, Soheila Shekari, Seyed Ali Askarpour, Naeemeh Hassanpour Ardekanizadeh, Mina Esmaeili, Morteza Abdollahi, Saeid Doaei, Sara Khoshdooz, Marjan Ajami, Maryam Gholamalizadeh

**Affiliations:** 1 Department of Community Nutrition, School of Nutritional Sciences and dietetic, Tehran University of Medical Sciences, Tehran, Iran; 2 School of Nutritional Sciences and Dietetics, Tehran University of Medical Sciences, Tehran, Iran; 3 Food Security Research Center, Department of Community Nutrition, School of Nutrition and Food Science, Isfahan, Iran; 4 Department of Nutrition Research, National Nutrition and Food Technology Research Institute, Shahid Beheshti University of Medical Sciences, Tehran, Iran; 5 Department of Nutrition, Science and Research Branch, Islamic Azad University, Tehran, Iran; 6 Division of Food Safety and Hygiene, Department of Environmental Health Engineering, School of Public Health, Tehran University of Medical Sciences, Tehran, Iran; 7 Torbat Jam Faculty of Medical Sciences, Torbat Jam, Iran; 8 Department of Nutrition Research, National Nutrition and Food Technology Research Institute, School of Nutrition Sciences and Food Technology, Shahid Beheshti University of Medical Sciences, Tehran, Iran; 9 Social Determinants of Health Research Center, and National Nutrition and Food Technology Research Institute, Faculty of Nutrition Sciences and Food Technology, Shahid Beheshti University of Medical Sciences, Tehran, Iran; 10 Department of Community Nutrition, National Nutrition and Food Technology Research Institute, Faculty of Nutrition Sciences and Food Technology, Shahid Beheshti University of Medical Sciences, Tehran, Iran; 11 Guilan University of Medical Sciences, Rasht, Iran; 12 Department of Food and Nutrition Policy and Planning Research, National Nutrition and Food Technology Research Institute, Shahid Beheshti University of Medical Sciences, Tehran, Iran; 13 Cancer Research Center, Shahid Beheshti University of Medical Sciences, Tehran, Iran

**Keywords:** Cardiovascular diseases, Ischemic heart disease, Dietary fats, Fatty acids

## Abstract

**Background:**

This study aimed to investigate the association between different types of dietary fats with ischemic heart disease (IHD).

**Methods:**

This case–control study was conducted on 443 cases and 453 controls aged 40–80 years in Tehran, Iran. The semi-quantitative 237-item food frequency questionnaire (FFQ) was used to assess the amount of food intake. Nutritionist IV was applied to test the amount of consumption of dietary fats.

**Results:**

The case group had a lower intake of docosahexaenoic acid (DHA) (11.36 ± 12.58 vs. 14.19 ± 19.57, *P* = 0.01) than the control group. A negative association was found between IHD and DHA (OR 0.98, CI 95% 0.97–0.99, *P* = 0.01). No significant association was observed between IHD with the intake of cholesterol, trans fatty acids (TFA), saturated fatty acids (SFA), monounsaturated fatty acids (MUFA), polyunsaturated fatty acids (PUFA), eicosatetraenoic acid (EPA), and α-Linolenic acid (ALA).

**Conclusion:**

It was found that DHA may reduce the risk of IHD, whereas there was no significant association between other types of dietary fats with the odds of IHD. If the results of this study are confirmed in future research, a higher intake of DHA in diet can be recommended as a strategy to prevent IHD events.

## Introduction

Among non-communicable illnesses, cardiovascular diseases (CVDs) account for more than 70% of the global mortality [1]. Ischemic heart disease (IHD), also known as coronary artery disease (CAD) or coronary heart disease (CHD), is the most prevalent type of CVDs, produced by an is related to the creation of atherosclerosis [2]. According to the Global Burden of Disease (GBD) study, IHD affects about 126 million people which is equal to 1.72% of the world’s population (1655 per 100,000). IHD was reported to be responsible for 9 million fatalities worldwide [3]. In Iran, the incidence rate reached 829.1 new cases (719.9–945.2) per 100,000 persons in 2019 [4]. Lifestyle management including having a healthy diet and proper physical activity, and also treatment of risk factors such as hypertension, diabetes, and lipid disorders may prevent IHD [5].

The studies on the link between IHD and dietary fats reported contradictory results. For example, in China, dietary fat consumption grew from 15.9% of calories in 1982 to 21.1% in 1990 and in the meantime IHD incidence and death likewise increased [6]. An unmatched case–control study which was conducted in Ethiopia indicated that poor diets such as cooking with palm oil and consuming fewer fruits and vegetables were related to IHD [7]. Some types of fatty acids were reported to be associated with IHD [8]. For example, epidemiological studies found a link between *n*−3 fatty acid consumption and a lower risk of IHD. This impact may be caused by changes in eicosanoid production and cholesterol levels [9]. Some intervention studies have not found that *n*−3 fatty acids can reduce the risk of IHD, although monounsaturated fatty acids (MUFAs) were identified to have beneficial effects [10].

Furthermore, recent studies found that saturated fatty acids (SFAs) boost IHD risk, whereas poly unsaturated fatty acids (PUFA) may decrease the risk of IHD [11]. High PUFA/SFA rates in diets may reduce IHD risk by affecting serum cholesterol levels and blood pressure [12]. Long chain omega-3 polyunsaturated fatty acids (PUFAs) may also reduce inflammation and platelet aggregability and improve endothelial function and atherosclerotic plaque stability [13]. To our knowledge, no research assessed the connection between dietary fatty acids consumption with IHD in Middle Eastern diets which may differ substantially from Western norms. So, the current study was done to investigate the association between dietary fat intake and the risk of IHD among Iranian adults.

## Methods

### Data collection

This is a population-based case–control study conducted in Tehran, Iran, between February 2021 and January 2022. The cases (*n* = 453) with a medical record of stable IHD confirmed by the physician were selected using a consecutive sampling method. Individuals free of IHD who refer to the same hospital for general check-ups were chosen as the control group (*n* = 453). The study's sample size was determined using EPI online software [14].

First, the purpose of the research and how it will be carried out were explained to all participants and written consent forms were collected. Professional interviewers gathered patients' general information on medical history, anthropometric indices, physical activity, and food intake. The ethics committee of the Shahid Beheshti University of Medical Sciences confirmed the study protocol (Code: IR.SBMU.NNFTRI.REC.1400.030).

Inclusion criteria for the case group were patients with IHD, no more than 3 months have passed since the diagnosis of the disease, written consent to participate, and age between 40 and 80. The inclusion criteria for the control group were the absence of IHD and an age range of 40 to 80 years. The non-entry criteria for were a history of diseases affecting food intake including mental illnesses, cancer, and malignant diseases. Exclusion criteria were not completion of the study (*n* = 10) and adherence to a diet, medication or supplement that interferes with the amount of dietary fat intake (*n* = 10). A total of 433 cases and 453 controls were included in final analyses.

### Anthropometric indices

The individual was weighed in minimal clothing and bare feet using a digital scale (SECA, Hamburg, Germany) accurate to the nearest 0.1 kg. Height was taken using a stadiometer mounted on the wall, and the result was accurate to within 0.5 cm. Body mass index (BMI) was determined using the following formula: BMI = weight/height^2^ (kg/m^2^).

### Physical activity

A short form of the International Physical Activity Questionnaire (IPAQ) was used to measure physical activity levels. The IPAQ consisted of seven questions. Overall, the questions assessed the number of days and minutes spent participating in light and heavy activities and the average time spent walking and sitting during the previous seven days. The metabolic equivalent of the task (MET) was used to measure and compare participants' levels of activity.

### Pathologic and biochemical assessment

In this study, diagnostic categories associated with clinical diagnosis (CD) based on the International Classification of Diseases 10 (ICD-10) (CD-ICD-10) were used to identify hospitalized IHD patients [15]. Biochemical and pathological measurements were collected to compare the health status of two groups.

### Assessment of dietary intake

A validated semi-quantitative 237-item food frequency questionnaire (FFQ) was used to assess the amount of food intake and the form of consumption of various foods [16]. The FFQ data were scaled down to grams and the participants’ intake of total fat, cholesterol, trans fatty acids (TFA), SFA, MUFA, PUFA, α-Linolenic acid (ALA), eicosapentaenoic acid (EPA), and docosahexaenoic acid (DHA) were measured using Nutritionist IV.

### Statistical analysis

Quantitative information was described using mean and standard deviation and qualitative information was described using number and percentage, which were compared between the groups using the independent t test and chi-square test, respectively. The IHD status was considered as the dependent variable and dietary fat consumption was considered as the independent variable in the logistic regression method, which was used in different models to control the confounding factors. The data were analyzed using SPSS, and a P value < 0.05 was considered statistically significant.

## Results

Characteristics of the participants are presented in Table 1. The cases had lower red blood cell (RBC) (4.86 ± 1.66 vs. 4.96 ± 1.52, P < 0.01) and higher white blood cell (WBC) (6.67 ± 0.53 vs. 6.32 ± 0.56, P < 0.01) and fasting blood sugar (FBS) (121.11 ± 37.36vs. 107.96 ± 43.20, P < 0.01) than the controls. There was no significant difference in terms of body mass index (BMI), smoking, drinking alcohol, right systolic blood pressure (SBP1), right diastolic blood pressure (DBP1), hemoglobin (Hgb), hematocrit test (HCT), mean corpuscular volume (MCV), mean corpuscular hemoglobin (MCH), mean cell hemoglobin concentration (MCHC), high-density lipoprotein cholesterol (HDL-C), and high-density lipoprotein cholesterol (LDL-C).

**Table 1:** Characteristics of the participants

	Cases (*n* = 433)	Controls (*n* = 453)	P
Age (Year)	55.59	54.67	0.11
Physical activity (METs-min/week)	37.53 ± 7.72	38.01 ± 8.38	0.39
Height (Cm)	161.48	161.03	0.46
Weight (Kg)	74.38	72.64	0.06
BMI (Kg/m^2^)	28.54	28.06	0.13
Smoking (n, %)	108 (24.83)	87 (19.21)	0.06
Male (n, %)	208 (47.92)	204 (45.03)	0.40
Use alcohol (n, %)	38 (8.74)	45 (9.94)	0.48
Right SBP1 (mmHg)	114.47 ± 16.63	114.51 ± 17.37	0.87
Right DBP1(mmHg)	71.950 ± 10.40	71.96 ± 10.64	0.84
WBC ($\times$ 10^3^ $\mu l$)	6.67 ± 0.53	6.32 ± 0.56	< 0.01
RBC ($\times$ 10^3^ $\mu l$)	4.86 ± 1.66	4.96 ± 1.52	< 0.01
Hgb (g/dl)	13.99 ± 1.53	14.08 ± 1.55	0.43
HCT (%)	41.04 ± 4.14	41.36 ± 4.29	0.17
MCV (fl)	84.87 ± 5.81	85.002 ± 5.72	0.80
MCH (pg)	28.95 ± 2.59	28.97 ± 2.54	0.85
MCHC (g/dl)	34.08 ± 1.43	34.05 ± 1.41	0.16
PLT ($\times$ 10^3^ $\mu l$)	283.75 ± 67.55	276.68 ± 68.09	0.20
FBS (mg/dl)	121.11 ± 37.36	107.96 ± 43.20	< 0.01
BUN (mg/dl)	13.58 ± 3.75	13.89 ± 3.82	0.09
Cr (mg/dl)	1.08 ± 0.27	1.10 ± 0.216	0.68
TG (mg/dl)	148.33 ± 109.08	144.51 ± 95.82	0.28
TC (mg/dl)	192.03 ± 40.39	191.26 ± 40.12	0.83
SGOT (U/L)	19.97 ± 7.39	20.40 ± 10.05	0.09
SGPT (U/L)	21.95 ± 13.52	22.29 ± 16.61	0.09
ALP (U/L)	222.07 ± 68.79	222.21 ± 67.23	0.67
HDL-C (mg/dl)	52.42 ± 10.54	52.35 ± 10.69	0.88
LDL-C (mg/dl)	110.16 ± 34.06	110.40 ± 33.43	0.81

Body mass index = BMI, Right systolic blood pressure = SBP1, Right diastolic blood pressure = DBP1, white blood cell = WBC, red blood cell = RBC, hemoglobin = Hgb, hematocrit test = HCT, mean corpuscular volume = MCV, mean corpuscular hemoglobin = MCH, mean cell hemoglobin concentration = MCHC, platelet count test = PLT, fasting blood sugar = FBS, blood urea nitrogen = BUN, creatinine = Cr, triglyceride = TG, total cholesterol = TC, serum glutamic oxaloacetic transaminase = SGOT, serum glutamic pyruvic transaminase = SGPT, alkaline phosphatase = ALP, high-density lipoprotein cholesterol = HDL-C, and high-density lipoprotein cholesterol = LDL-C

A comparison of dietary intake among the case and control groups is presented in Table 2. The case group had a lower intake of DHA (11.36 ± 12.58 vs. 14.19 ± 19.57, *P* = 0.01) than the control group. No significant difference was found in terms of the intake of calorie, protein, total fat, carbohydrate, micronutrients, and other types of dietary fats.

**Table 2:** Dietary nutrient intake among the ischemic heart disease (IHD) and the control groups

	Cases (*n* = 433)	Controls (*n* = 453)	P*
Energy (kcal)	2482.58 ± 768.69	2511.78 ± 799.43	0.25
*Macronutrients*			
Protein (g/d)	78.37 ± 25.76	78.97 ± 26.36	0.45
Fat (g/d)	64.39 ± 24.79	64.59 ± 25.71	0.23
Carbohydrate (g/d)	409.62 ± 135.34	415.49 ± 140.59	0.19
Fiber (g/d)	27.53 ± 10.37	27.23 ± 10.12	0.71
Sugar (g/d)	138.95 ± 63.78	139.21 ± 64.15	0.84
Caffeine (g/d)	191.52 ± 128.76	139.21 ± 64.16	0.24
Galactose (g/d)	0.192 ± 0.188	0.191 ± 0.216	0.58
*Micronutrients*			
Calcium (mg/d)	912.24 ± 328.5	918.67 ± 329.5	0.83
Iron (mg/d)	13.38 ± 4.73	344.82 ± 110.76	0.80
Magnesium (mg/d)	344.8 ± 110.76	343.98 ± 110.69	0.83
Phosphorus (mg/d)	1198.2 ± 389.77	1199.44 ± 398.51	0.42
Potassium (mg/d)	3674.27 ± 1278.79	3642.93 ± 1257.57	0.74
Sodium (mg/d)	4554.7 ± 2054.61	4633.84 ± 2088.36	0.79
Zinc (mg/d)	10.08 ± 3.35	10.08 ± 3.42	0.44
Copper (mg/d)	1.83 ± 0.71	1.83 ± 0.66	0.66
Fluoride (mg/d)	3573.2 ± 2394.6	3576.44 ± 2342.34	0.23
Manganese (mg/d)	5.65 ± 1.95	5.66 ± 1.93	0.92
Selenium (mg/d)	55.78 ± 29.63	54.66 ± 28.13	0.66
Vitamin A (IU)	8692.01 ± 5640.81	8720.66 ± 5552.12	0.62
Retinol (mg/d)	338.25 ± 394.44	333.88 ± 281.89	0.55
Vitamin A ($\mu$ g/d)	715.08 ± 508.06	713.19 ± 425.37	0.96
$\beta$-Carotene ($\mu$g/d)	4019.38 ± 2739.06	4044.64 ± 2746.80	0.41
$\alpha$-Carotene (μg/d)	665.44 ± 838.02	685.43 ± 856.74	0.21
Vitamin E ($\alpha$ -tocopherol)	7.24 ± 3.28	7.14 ± 3.22	0.65
Vitamin D (IU)	42.91 ± 27.26	42.96 ± 27.10	0.29
Vitamin D_2,_ D_3_ ($\mu$ g/d)	1.21 ± 0.703	1.21 ± 0.69	0.24
Vitamin C (ascorbic acid)	143.73 ± 83.78	139.96 ± 78.97	0.45
Vitamin B_1_ (mg/d)	1.62 ± 0.59	1.64 ± 0.60	0.82
Vitamin B_2_ (mg/d)	1.76 ± 0.66	1.74 ± 0.626	0.54
Vitamin B_3_ (mg/d)	18.16 ± 6.53	18.17 ± 6.51	0.68
Vitamin B_5_ (mg/d)	5.93 ± 1.90	5.90 ± 1.92	0.45
Vitamin B_6_ (mg/d)	9.77 ± 5.91	10.04 ± 4.91	0.51
Vitamin B_9_ ($\mu$ g/d)	381.07 ± 138.89	377.18 ± 130.77	0.36
Vitamin B_12_ ($\mu$ g/d)	6.07 ± 6.43	5.87 ± 4.50	0.26
Vitamin K (mg/d)	164.5 ± 102.65	165.39 ± 100.9	0.54
*Fatty acids*			
Cholesterol (g/d)	259.8 ± 122.1	260.01 ± 120.05	0.56
Trans fatty acids (TFA) (g/d)	0.241 ± 0.27	0.24 ± 0.27	0.58
Saturated fatty acids (SFA) (g/d)	25.09 ± 11.16	25.21 ± 11.43	0.70
Monounsaturated Fatty acids (MUFA) (g/d)	20.11 ± 8.19	20.06 ± 8.43	0.26
Polyunsaturated fatty acids (PUFA) (g/d)	11.76 ± 4.72	11.77 ± 4.94	0.18
Docosahexaenoic acid (DHA) (mg/d)	113.6 ± 12.58	141.9 ± 19.57	0.01
Eicosatetraenoic acid (EPA) (mg/d)	460.75 ± 0.317	452.04 ± .0.319	0.71
$\alpha -$ Linolenic acid (ALA) (g/d)	7.15 ± 4.58	7.22 ± 3.78	0.69

*Independent sample t test

The association between IHD and different types of dietary fats is shown in Table 3. A negative association was found between IHD and DHA (OR 0.98, CI 95% 0.98, CI 95% 0.97–0.99, *P* = 0.01). The association remained significant after adjustment for age, and gender (Model 2), additional adjustment for total fat and energy (Model 3), additionally adjustment for BMI (Model 4), and further adjustment for smoking, use alcohol, and physical activity (Model 5).

**Table 3:** Logistic regression of the association between ischemic heart disease (IHD) and dietary fats

	DHA		SFA		MUFA		PUFA		Trans	
	OR (CI 95%)	P	OR (CI 95%)	P	OR (CI 95%)	P	OR (CI 95%)	P	OR (CI 95%)	P
Model 1	0.98 (0.97–0.99)	0.01	0.99 (0.97–1.01)	0.52	1.01 (0.98–1.03)	0.47	0.97 (0.94–0.99)	0.43	1.02 (0.97–1.07)	0.41
Model 2	0.98 (0.97–0.99)	0.01	0.99 (0.98–1.01)	0.81	1.002 (0.97–1.02)	0.91	0.97 (0.94–1.01)	0.19	1.02 (0.97–1.07)	0.32
Model 3	0.98 (0.97–0.99)	0.03	0.99 (0.98–1.01)	0.90	1 (0.97–1.02)	0.99	0.98 (0.94–1.01)	0.21	1.02 (0.97–1.07)	0.31
Model 4	0.98 (0.97–0.99)	0.03	0.99 (0.98–1.01)	0.85	1.002 (0.97–1.03)	0.91	0.97 (0.94–1.009)	0.16	1.02 (0.97–1.08)	0.26
Model 5	0.98 (0.97–0.99)	0.04	1.05 (1.001–1.10)	0.44	1.04 (1–1.10)	0.45	1.02 (0.96–1.08)	0.42	1.02 (0.97–1.08)	0.26

**Fig. 1:**
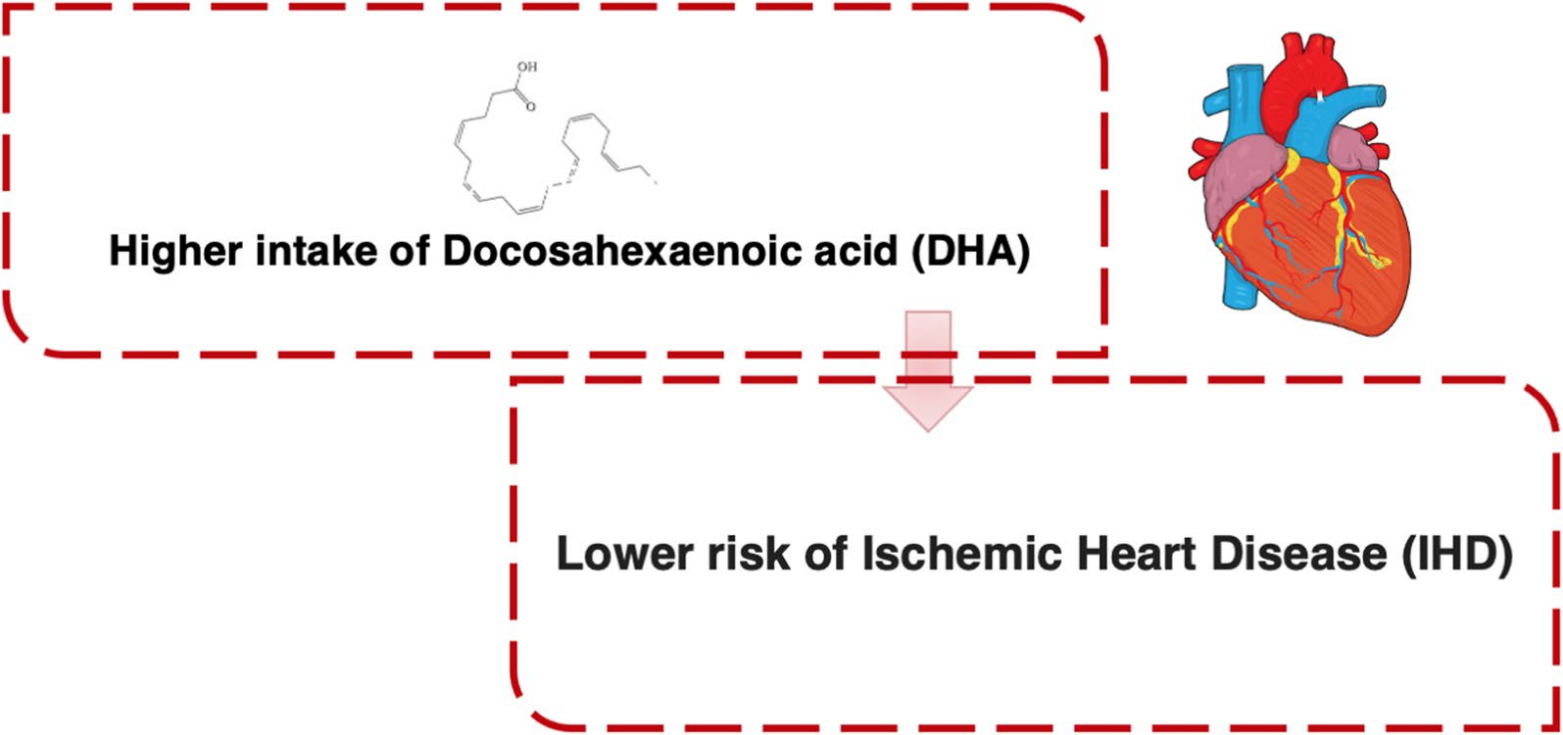
The association between Ischemic Heart Disease (IHD) with dietary fatty acids

Model 1: crude, Model 2: adjusted for Age at interview, Gender, Model 3: Additionally adjusted for total fat and energy, Model 4: Additionally adjusted for BMI, Model 5: Further adjusting for Smoking, Use Alcohol, and physical activity

age and sex, and additional adjustment for dietary calorie and total fat, BMI, smoking, and drinking alcohol. No significant relationship was observed between the intakes of cholesterol, SFA, MUFA, with IHD (Fig. 1).

## Discussion

The present study found that consuming DHA may reduce the risk of IHD by 2%. However, we found no evidence of an association between IHD with other dietary fat such as SFA, TFA, MUFA, PUFA, ALA, and EPA.

In line with the present result, some previous studies reported that *n*−3 FAs may have a beneficial impact on the risk of cardiovascular death in patients with IHD [17]. Several studies showed that omega-3 fatty acids, especially DHA (a prominent kind of *n*−3 FA in fish oil) and ALA (the most frequent type of *n*−3 FAs in plant oils), may have a significant role in prevention of cardiovascular diseases [18, 19]. One possible mechanism is that DHA reduces blood pressure in both the normotensive [20, 21] and hypertensive [22] individuals [23, 24]. Also, considering the association between left ventricular (LV) function and atherosclerotic coronary disease in patients with IHD [25], an experimental study showed that supplementation with DHA substantially decreased LV dysfunction after 12 weeks [26]. Since DHA is known to decrease smooth muscle cell proliferation in cell culture, the mechanism of DHA-mediated athero-protection may be related to blood flow patterns and wall shear stress (WSS) that influence inflammation both locally and at distant atheroprone sites [17, 27, 28]. Furthermore, the acute coronary syndromes (ACS) caused by a rupture of the plaque’s fibrous cap have both been linked to DHA deficiency-induced inflammation [28]. Despite the proposed effect of DHA on IHD, some studies reported contradictory result. For example, Xu et al. reported opposite results in a randomized trial [29]. Also, another RCT showed that treatment with n-3 PUFAs (1 g DHA + EPA daily, with a median of 5 years of follow-up) did not reduce the incidence of cardiovascular events in patients with multiple cardiovascular risk factors [30].

On the other hand, higher intake of SFA is frequently reported to be a risk factor of IHD as a pro-atherogenic factor [31]. However, this study failed to identify a significant relation, other studies indicated that there is SFA had an adverse effect on IHD by increasing the total blood cholesterol concentration [32] and eventually increase the risk of ischemic stroke [33–35]. In line with the present study, some studies found no association between IHD and SFA [36, 37]. For example, Praagman et al. in a cohort study showed that there is no relation between higher intake of SFA and risk of IHD and suggested that this association was determined by the length of the SFA chain and the source of the SFAs. A lower IHD risk was associated with higher intakes of the sum of butyric acid, capric acid, myristic acid, the sum of pentacyclic and margaric acids, and also with SFAs from dairy sources (milk and milk products, cheese, and butter) [38].

In addition, the present result failed to show evidence of the association of PUFA, MUFA, and TFA with IHD. A possible explanation for the present result is the consumption of MUFA and PUFA is low among the Iranian population [39]. Another possible reason is that certain foods that Iranians consume contain both TFA and PUFA and these two may counteract each other's effects in IHD [40].

The strengths of this study include a reasonable sample size, multiple adjustments for relevant confounders, evaluation of a less studied population, and using a validated FFQ with 237 items. However, this study had some limitations. First, this study was conducted among Iranian adults, so generalization to other populations should be made with caution. Second, although the association of IHD and dietary intake of DHA was statistically significant, the OR was closed to 1, which may indicate no clinically relationship. Further longitudinal studies are warranted to confirm these findings and prove whether providing DHA in the form of food or supplements can be used as a nutritional recommendation for patients at risk of IHD.

## Conclusion

In conclusion, it was found that DHA may reduce the risk of IHD, whereas no significant association between SFA, PUFA, MUFA, and TFA with the odds of IHD was realized. These findings need to be confirmed by future large-scale studies. If the results of this study are confirmed in future longitudinal studies, a higher intake of DHA in diet can be recommended as a strategy to prevent IHD events.

## Data Availability

Not applicable.
