# Correction to: Association of dietary fats with ischemic heart disease (IHD): a case–control study

**DOI:** 10.1186/s41043-024-00547-x

**Published:** 2024-04-16

**Authors:** Mobina Zeinalabedini, Maryam Ladaninezhad, Khadijeh Abbasi Mobarakeh, Anahita Hoshiar-Rad, Soheila Shekari, Seyed Ali Askarpour, Naeemeh Hassanpour Ardekanizadeh, Mina Esmaeili, Morteza Abdollahi, Saeid Doaei, Sara Khoshdooz, Marjan Ajami, Maryam Gholamalizadeh

**Affiliations:** 1 Department of Community Nutrition, School of Nutritional Sciences and dietetic, Tehran University of Medical Sciences, Tehran, Iran; 2 School of Nutritional Sciences and Dietetics, Tehran University of Medical Sciences, Tehran, Iran; 3 Food Security Research Center, Department of Community Nutrition, School of Nutrition and Food Science, Isfahan, Iran; 4 Department of Nutrition Research, National Nutrition and Food Technology Research Institute, Shahid Beheshti University of Medical Sciences, Tehran, Iran; 5 Department of Nutrition, Science and Research Branch, Islamic Azad University, Tehran, Iran; 6 Division of Food Safety and Hygiene, Department of Environmental Health Engineering, School of Public Health, Tehran University of Medical Sciences, Tehran, Iran; 7 Torbat Jam Faculty of Medical Sciences, Torbat Jam, Iran; 8 Department of Nutrition Research, National Nutrition and Food Technology Research Institute, School of Nutrition Sciences and Food Technology, Shahid Beheshti University of Medical Sciences, Tehran, Iran; 9 Social Determinants of Health Research Center, and National Nutrition and Food Technology Research Institute, Faculty of Nutrition Sciences and Food Technology, Shahid Beheshti University of Medical Sciences, Tehran, Iran; 10 Department of Community Nutrition, National Nutrition and Food Technology Research Institute, Faculty of Nutrition Sciences and Food Technology, Shahid Beheshti University of Medical Sciences, Tehran, Iran; 11 Guilan University of Medical Sciences, Rasht, Iran; 12 Department of Food and Nutrition Policy and Planning Research, National Nutrition and Food Technology Research Institute, Shahid Beheshti University of Medical Sciences, Tehran, Iran; 13 Cancer Research Center, Shahid Beheshti University of Medical Sciences, Tehran, Iran

**Correction to:*J Health Popul Nutr*43**, 19 (2024).

10.1186/s41043-023-00489-w.

Following publication of the original article [1], the authors identified an error in the author name of Mobina Zeinalabedini.

The incorrect author name is: Zeinolabedin Amini Sabegh.

The correct author name is: Mobina Zeinalabedini.

The author group has been updated above and the original article [1] has been corrected.

## References

[1] Sabegh ZA, Ladaninezhad M, Mobarakeh KA (2024). Association of dietary fats with ischemic heart disease (IHD): a case–control study. J Health Popul Nutr.

